# Successful management of ivermectin-induced blindness in an African lion (*Panthera leo*) by intravenous administration of a lipid emulsion

**DOI:** 10.1186/s12917-015-0603-6

**Published:** 2015-11-26

**Authors:** Muhammad Saqib, Ghazanfar Abbas, Mudassar Niaz Mughal

**Affiliations:** Department of Clinical Medicine and Surgery, Faculty of Veterinary Sciences, University of Agriculture Faisalabad, 38040 Punjab, Pakistan; The Equine Center, 4850 Davenport Creek Road, San Luis Obispo, CA 93401 USA; State key of agricultural microbiology, Hzauhong Agriculture university, 430070 Wuhan, China

**Keywords:** Ivermectin, Lion, Blindness, Lipid emulsion

## Abstract

**Background:**

Ivermectin is widely used in veterinary practice for the treatment of ecto- and endo-parasites. In wildlife, an extra-label use this parasiticide is sometimes associated with toxicity. Different treatment regimens have been used in ivermectin toxicosis. The present report describes a successful reversal of ivermectin toxicity by intravenous administration of a commercially available lipid emulsion in a captive African lion (*Panthera leo*).

**Case presentation:**

A 2-year old captive African lion (*Panthera leo)* weighing ~130 kg was presented with acute neurological impairment and bilateral blindness that had developed 24 h after ivermectin exposure. The animal was treated with a commercially available lipid emulsion along with supportive therapy and experienced complete recovery.

**Conclusion:**

To our knowledge, this is the first case report of the use of lipid emulsion in the management of ivermectin induced blindness in an African lion and it appears that intravenous lipid emulsion may be an effective therapy in ivermectin toxicity in lions. Further testing in expanded clinical trials is clearly warranted.

## Background

Ivermectin (22, 23-dihydroavermectin B1_a_ + 22, 23-dihydroavermectin B1_b_) is a semi-synthetic macrocyclic lactone belonging to avermectin family with a wide range of antiparasitic activity [1]. This in an injectable form is approved for use in ruminants only [2, 3]. However, an extra-label use is common and has had documented usage for the treatment of parasites in various other animal species [4]. In species including lions, an extra-label use of ivermectin has reportedly been made for the control and treatment of ectoparasites, including ticks, mites, flies and some endoparasites such as intestinal round worms and systemic filaroides [5]. The mechanism of action of ivermectin is multifaceted and entails potentiation of the discharge of the GABA (an inhibitory neurotransmitter) in the peripheral nervous system of the parasitic invertebrates, resulting in an influx of chloride ions, hyperpolarization of neuronal membranes that ensues in neuronal dysfunction and flaccid paralysis [6–8]. In mammals, where GABA receptors are present only in the central nervous system, the *p*-glycoprotein pump encoded by the multidrug resistance gene (*MDR1,* also known as ABCB1-∆) is present in an intact blood-brain barrier and protects from the neurotoxic effects of ivermectin [8, 9]. Ivermectin toxicity has been reported in several species of animals including dogs, cats, cattle, horses, pigs, frogs and chelonians [7]. Nevertheless, ivermectin toxicity-induced blindness in lions (*Panthera leo*) is an uncommonly reported condition and to date, only a single report has been published [5]. To the best of our knowledge, this is the first documented report of ivermectin toxicity-induced blindness in a lion successfully treated by using intravenous lipid emulsion (ILE).

## Case presentation

A 2-year old captive African lion (*Panthera leo)* weighing ~130 kg was admitted to Veterinary Medical Teaching Hospital (VMTH), University of Agriculture Faisalabad, Pakistan for the treatment of acute neurological impairments, including ataxia, apparent hallucinations, generalized seizures, disorientation and bilateral blindness. This impairment had occurred 24 h after the oral administration of an overdose (>10-fold) of ivermectin (Tab. Mectimite™, Pharama Health, Pakistan). Ivermectin dosage was incorrectly determined; instead of receiving the recommended prophylactic dose (0.3 mg/kg), the animal received 3 mg/kg. The lion was strictly confined to a cage and fed with raw beef and milk. Vaccination status of the lion was recent and included those against rhinotracheitis virus, calicivirus and panleucopenia virus. At presentation on VMTH, the lion was in a stuporous condition but responded to pain stimuli. Clinical examination revealed decreased rectal temperature (36.6 °C), bradycardia (50 beats/min), bradypnea (15 breaths/min) and slightly increased salivation. Superficial and peripheral arteries (femoral and brachial) were devoid of any detectible pulse and had a prolonged capillary refill time (>5 s), along with poor jugular vein filling. Extremities were cold to touch and oral mucosa was pale and slightly tacky. Ophthalmic examination indicated diminished menace response, direct and indirect pupillary and palpebral reflexes in both eyes, although the corneal reflex was present and spontaneous horizontal nystagmus was evident in both eyes. Defecation and urination were normal. Intermittent twitching of the muscles surrounding shoulder and gluteal regions along with periodic jerking of the head were recorded. Both superior and inferior lips were flaccid and had proprioceptive deficits along with evident limb weakness in all limbs. Significant hematologic findings included microcytic normochromic anemia and leukocytosis composed of monocytosis and neutrophilia (Table 1). Serum biochemical alterations included elevated levels of gamma glutamyl transferase (GGT), alkaline phosphatase (ALP), creatinine and total proteins (TP) including albumin and globulin along with decreased serum calcium and glucose (Table 2). Thoracic radiographs and fecal examination did not point to any significant abnormality. Initial therapeutic management consisting of intravenous supply of isotonic crystalloid solution (@ 30 ml/kg, b.wt; Infusion Ringolact™, Otsuka, Pakistan) supplemented with 2.5 % glucose (5 ml/kg/hr) along with diazepam (@ 0.1 mg/kg, b.wt; Inj. Valium™, Roche Pakistan) was instituted. Activated charcoal (@1 g/kg, b.wt; Cap. Karbon™, Neo-Madix Pharma, Pakistan) was administered using orogastric tube. After 2 h of treatment, the CRT, peripheral pulse quality and jugular vein filling had improved and the lion urinated. Vital parameters recorded at this time included a slightly decreased rectal temperature (37 °C), bradycardia (60 beats/min) and severe respiratory depression (10 breaths/min). The extremities of the lion were bandaged and the animal was placed beneath an infrared heat lamp on a forced-air heating blanket for 30 min until the rectal temperature raised to 38.2 °C. The animal was then able to maintain normal body temperature. In view of severe respiratory depression, intubation was performed and manual positive inspiratory pressure ventilation was supplied with the aid of self-inflating manual resuscitator (AMBU-bag China) by using room air at 20 breaths/min. Atropine sulfate (@ 0.02 mg/kg b.wt, IV; Inj. Atrosol™, Indus Pharma, Pakistan) was administered to address bradycardia. In spite of this treatment, the lion remained comatosed for 8 h after presentation at VMTH. Aware of a successful treatment in a previous ivermectin toxicosis case in a felid [10], we administered two repeated doses (6 h apart) of neostigmine methylsulphate (@ 0.02 mg/kg, IV; Inj. Neostigmine™, Goodman International, Pakistan to antagonize the ivermectin-induced effects on GABA receptors. Fluid therapy was continued with an isotonic crystalloid solution (@ 30 ml/kg, b.wt; Infusion Ringolact, Otsuka, Pakistan). No improvement in neurological status was observed till 36 h of treatment. The animal vomited a small amount of digested food containing activated charcoal. A small amount of vomitwas also seen in the endotracheal tube indicating that aspiration had occurred. Immediately, the endotracheal tube was changed and treatment with ceftriaxone sodium (@ 20 mg/kg, b.wt., IV, q12h; Inj. Oxidil™; Sami Pharmaceuticals, Pakistan) and dexamethasone (@ 0.5 mg/kg, b.wt, IV q12 h; Inj. Dcadran®, OBS Pakistan) instituted. The stomach was lavaged with approximately 3 l of water to remove any residual food and activated charcoal. The vital physiological parameters recorded at this time included normal rectal temperature (38 °C), severe bradycardia (30 beats/min) and bradypnea (15 breaths/min). Point-of-care testing revealed similar findings as observed initially at the time of admission (Tables 1 and 2).

**Table 1:** Hematology profile of the lion (*Panthera leo*) affected with ivermectin intoxication

Parameter	Presenting Values					Reference Values^a^
	At Presentation	36 hours of presentation	72 hours of presentation	92 hours of presentation	After 2 weeks	
Red blood cells (× 10^12^ g/L)	3.71	3.70	3.83	3.69	5.72	5.10-11.70
Packed cell volume (L/L)	0.076	0.073	0.081	0.065	0.201	0.251- 0.520
Hemoglobin (g/L)	20.1	19.7	21	18.6	47.9	44-230
Mean corpuscular volume (fL)	20.4	19.7	21.1	17.6	70.1	29.9-76
Mean corpuscular hemoglobin (pg)	11.9	13.7	11.5	14.8	17.9	11.2-27.2
Mean corpuscular hemoglobin concentration (g/L)	264	269	259	286	238	231-428
White blood cells (× 10^9^/L)	50.3	49.7	52.7	47.9	18.5	5.50-29.40
Neutrophils (× 10^9^/L)	36.29	35.27	37.54	35.23	6.69	0.000-6.69
Lymphocytes (× 10^9^/L)	0.009	0.008	0.01	0.01	7.64	0.007-8.340
Monocytes (× 10^9^/L)	13.70	13.52	14.45	12.06	2.761	0.000-2.912
Eosinophils (× 10^9^/L)	0.3	0	0.7	0.59	1.41	0.000-1.575

^a^International Species Information System (ISIS) Physiological Reference Values for *Panthera leo*

**Table 2:** Serum biochemistry profile of the lion (*Panthera leo*) affected with ivermectin intoxication

Parameter	Presenting Values					Reference Values^a^
	At Presentation	36 hours of presentation	72 hours of presentation	92 hours of presentation	After 2 weeks	
Aspartate Aminotransferase (U/L)	72	95	92	90	78	9-171
Alanine Aminotransferase (U/L)	102	98	97	90	101	19-161
Alkaline Phosphatase (U/L)	210	200	197	153	97	0-166
Gamma glutamyl transferase (U/L)	108	99	82	47	12	0-10
Total Proteins (g/L)	160	145	120	102	87	56-94
Albumin (g/L)	60.3	58	49	40	27	29-32
Globulin (g/L)	99.7	87	71	62	60	22-61
Creatinine (μmole/L)	403	397	390	367	223	0-389
Blood Urea Nitrogen (mmol/L)	109.8	100.6	75.41	45.78	22.67	4.641-25.35
Glucose	1.01	1.45	1.23	2.78	7.79	3.710-15.37
Calcium (mMol/L)	0.23	0.59	0.27	1.08	2.90	2.03-3.03

^a^International Species Information System (ISIS) Physiological Reference Values for *Panthera leo*

Despite this treatment, the animal continued to deteriorate, so treatment with neostigmine was substituted with ILE comprising of 20 % soybean oil in water (Intralipid® 20 %™, Fresenius Kabi (Schweiz AG). Because there is no recommendation available for the use of ILE for the treatment of lipophilic drug related toxicities in lions, the dosage was adjusted based on previous literature wherein lipid emulsions were administered to other felids [11] and to humans [12]. Initially, a bolus of 1.5 ml/kg (total = 200 ml) followed by 0.25 ml/kg/min for 30 min was administered intravenously by cephalic catheter and the animal was closely monitored for cardinal parameters and neurological status during and after the treatment. After approximately one hour post-completion of the lipid emulsion, the lion exhibited a slight progress of the pupillary light reflex in both eyes and the nystagmus stopped. The animal attempted to stand on his legs but was initially unsuccessful. After ~72 h of ivermectin exposure, the lion started partially regaining weight on his legs independently and appeared to visually follow and paw at imaginary objects. The animal ran into obstacles under scotopic and photopic state. Since no additional clinical improvement was observed, the animal received a second dose of ILE at the dose rate of 0.5 ml/kg/min for 30 min after 20 h of first dose (92 h after exposure to ivermectin) and again the continuous monitoring of cardinal parameters along with neurological status was performed.

Immediately after the completion of second dose of ILE, the lion was able to support his weight and remained standing for a long duration. Approximately, 1 h after completion of the second dose of ILE, eyesight appeared to improve as evidenced by successful navigation around the cage and pupillary light reflexes with menace responses evident in both eyes. The lion regained appetite reflux and appeared neurologically normal. Again the lion underwent blood collection for hematology and serum biochemistry, with results similar to those recorded on the first day of admission except for serum calcium and glucose (Tables 1 and 2). However, the cardinal parameters were normal. The lion was discharged on 4^th^ day after admission and his local veterinarian was advised to continue a treatment consisting of fluid therapy with isotonic crystalloid maintenance solution, antibiotics and corticosteroids for the next 3 days. After two weeks of treatment, the lion returned to VMTH for re-evaluation. All the laboratory tests were within normal reference ranges and no abnormal clinical and neurological signs were recorded even after 1 month follow up period. From all evidence presented, it appears likely that the subject lion had blindness due an ivermectin overdose and ILE treatment was instrumental in counteracting the ivermectin toxicosis.

## Discussion

The macrolides are divided into 2 classes’ including avermectin and milbemycin. The class avermectin includes ivermectin, doramectin, abamectin, eprinomectin and selamectin. However, moxidectin, nemadectin and milbemycin are the members of class milbemycine [13]. All these aforementioned antiparastic drugs have a wide range of safe and effective usages if prescribed as indicated on the labels. Because of highly lipophilic properties, ivermectin is well absorbed through parenteral, oral or topical route with an excretory rate of >90 % through feces and <2 % from urine. Ivermectin intoxication has been well documented in the veterinary literature and usually results from overdose or improper administration of the product intended for large animals. The severity of clinical signs associated with ivermectin intoxication depends upon the level of exposure and age of animals and includes lethargy, bradycardia, ataxia, hypersalivation, vomiting, muscular tremors, mydriasis, coma, obtundation, respiratory failure, apparent blindness and even death [2, 3, 14, 15]. Among dogs, a sub population of Collies and other related breeds are commonly susceptible to ivermectin intoxication due to homozygosity at for ABCB1-∆ locus Consequently, the defective *p*-glycoprotein in these dogs will be unable to protect the CNS from toxic dose of macrolides [16]. Young animals are more prone to the toxic effects of all the macrolides because they have an immature blood-brain barrier that is unable to keep avermectins out of the CNS [16].

Historically, no study has been conducted to assess the toxic level of ivermectin in lions, however, a what limited data that exist do not give a clear recommended therapeutic dose. Some studies in lions have successfully evaluated the anthelmintic activity of ivermectin against several nematodes at a dose rate of 0.3 mg/kg body weight [17]. However, a similar dose of ivermectin resulted in the onset of acute intoxication in a group of predisposed lions [5] and dogs [14, 18] suggesting further investigations of dosage and this drug should be used cautiously. Although, ivermectin toxicosis is well known in companion animals [19, 20], there is dearth of literature regarding ivermectin-induced blindness in lions. Few of the intoxicated animals may manifest apparent blindness with or without other clinical signs. We found published data documenting retinal lesions associated with presumptive ivermectin intoxication in 2 dogs in 1989 [21]. Epstein and Hollingsworth [22] also reported a case of apparent blindness in a Jack Russell Terrier subsequent to an apparent ivermectin overdose. A detailed ophthalmic examination of the affected dog carried out using slit lamp biomicroscopy, indirect ophthalmoscopy and electroretinography revealed diminished pupillary light reflex, menace response, dazzle reflex and retinal edema in both the eyes. The dog was treated with ILE and subsequently recovered. A similar case of bilateral blindness was also reported in a miniature mule foal [23]. The diagnosis was based on ophthalmic examination and electroretinography. The foal responded favorably to symptomatic treatment and supportive care. Unfortunately, in our case, slit lamp biomicroscopy and electroretiongraphy were not available so the diagnosis of apparent blindness was postulated on the basis of a known history of massive ivermectin overdose followed by findings of indirect and direct opthalmoscopy, hemato-biochemical analysis, clinical signs and response to neostigmine. Further confirmation by analysis of serum ivermectin levels could have been performed, but was deemed unnecessary because determination of ivermectin in the serum or plasma is not diagnostically fruitful because they would have only corroborated that the lion was treated with the ivermectin [24]. However, the concentration of the ivermectin in the brain tissue is more confirmatory and in mammals with an intact blood-brain barrier this concentration should be negligible.

The exact mechanism by which ivermectin induces blindness is yet to be determined. The published data on different animals suggest that the pathology of retina and some of its components especially adjacent optic nerves are involved in this process. The majority of cell types present inside the retina express GABAergic receptors and GABA is thought to be a key inhibitory neurotransmitter present inside the mammalian retina. It is speculated that if ivermectin passes the blood-retinal barrier (BRB), neurons present in the retina may be affected similarly to neurons within the CNS [25, 26]. Blindness associated with ivermectin intoxication is usually ephemeral and anecdotally, recovery is anticipated in 2-8 days [27], although the precise recovery time is unknown. Typically recovery is often prolonged and may take days to weeks [14].

The existing therapeutic recommendations for ivermectin intoxication include symptomatic treatment along with nursing care and nutritional support as required [28]. Treatment with physostigmine and neostigmine has reportedly resulted in transient clinical improvement in intoxicated animals, however, their use was discouraged due to several adverse reactions especially lacrimation, salivation and seizures. Additionally, these agents have short duration of action and require multiple doses to manage intoxication. In the subject of the present case report, the use of neostigmine seems justifiable by its successful use in a previous study [10]. Although the use of benzodiazepines for ivermectin intoxication is proscribed because of their GABA augmenting properties [29], however, in the current case its use was limited with an attempt to control hypersensitivity and tremors. The use of a corticosteroid (dexamethasone) in the current case was justified by its potential role in increasing blood glucose level and reducing the inflammatory response in aspiration pneumonia [30], by inhibiting activation of inflammatory cells, microvascular leakage and mucous formation. Although, in general practice, the reliable treatment recommendations for use of corticosteroids warrants executing randomized controlled clinical trials keeping in view certain key questions including dosage, frequency of administration and the potential side effects [30].

In the case presented here, the recorded serum biochemical alterations including elevated level of ALP and GGT might be attributed to hepatocellular damage as described previously in various other species including foals [23] and dogs (15). Moreover, hyperproteinemia and increased level of creatinine may be ascribed to dehydration. Elevated level of BUN is a key indicator of kidney malfunctioning. The hematological alterations observed in the affected lion including leukocytosis comprising of neutrophila and monocytosis is consistent with findings documented earlier [31] and probably are the result of underlying stress and hepatocellular injury. Furthermore, the documented microcytic normochromic anemia may be associated with iron deficiency secondary to ivermectin intoxication and decreased dietary intake as the lion was anorexic [32].

Previously, decreased levels of RBCs, PCV, Hb, lymphocytes, basophils and eosinophils along with increased neutrophils, band cells and monocytes have been observed in different toxicological studies on ivermectin [31–33]. Of these, sero-biochemical findings, elevated levels of GGT, ALP, creatinine, BUN are consistent with previous reports of ivermectin toxicity in animals [31, 33, 34]. Hepatocellular necrosis, renal tubular cell degeneration and pulmonary hemorrhages have been suggested as a potential cause of biochemical alterations in goats that received 10 times the standard dose of ivermectin [33].

ILE, also referred as lipoid emulsions, have been used since long as a component of parenteral nutrition for the treatment of organophosphate and local anesthetic drugs toxicities and as a vehicle for the transfer of several lipophilic drugs including etomidate, propofol, diazepam and paclitaxel [35]. Recently, ILEs have also been used as an antidote for ivermectin toxicity in various species of animals [15, 36, 37]. Previously, ILE has been successfully used to treat ivermectin intoxication in various breeds of dogs including Australian Shepherd [36], Jack Russell Terrier [22], Border Collie [16] as well as in a miniature Shetland pony [37]. Additionally, ILE has also been used to address moxidectin toxicosis in a puppy [28]. Generally, ILE is composed of either medium-chain triglycerides (MCTs) or long-chain triglycerides (LCTs) and sometime by combination of both. The most frequently used preparations of ILEs contain LCTs with a concentration of 10-30 % along with certain amount of glycerol and egg phospholipids [35]. The LCTs are comprised of free fatty acids including oleate, linolenate, palmitate, stearate and linoleate. ILEs can be obtained either from plant or marine sources. Among plant-based sources soybean oil is commonly used because it is a good source of essential fatty acids especially linoleate and linolenate [38].

The therapeutic uses of ILE for drug intoxication originated in studies in humans designed for investigating the metabolic effects of bupivacaine. The results of various substantial studies in animals have concluded that the adverse cardiovascular effects of toxic dose of bupivacaine could be ameliorated by the administration of ILE [39].

The precise mechanism behind antidotal action of ILEs is still unsolved but there are three proposed theories involved in treatment. The first and most widely accepted theory is the “lipid sink” theory, which postulates that after the infusion of any lipid solution, a lipid compartment is generated within the plasma that remains separated from the aqueous phase of the plasma. The offending drugs are withdrawn from the affected tissues of the body (e.g., CNS) into this lipid rich plasma phase and eventually excreted from the body [28]. This theory is bolstered by the results of various studies demonstrating the successful use of ILEs in the management of intoxication caused by the drugs having mechanism of action totally different from bupivacaine. ILEs have been used successfully for the treatment of lamogitrine, clomipramine, verapamil and buproprion intoxication in different animal models [40, 41].

The second proposed mechanism entails the boosting of cardiac energy supplies. During resting and non stressed phase of cardiac activity, fatty acids serve as a fuel for the production of ATP by cardiac myocytes. Some investigations have shown the beneficial effects of fatty acids during cardiac stress and thus ILE improves its efficiency subsequent to any drug intoxication associated pathological insult especially ischemia and necrosis [42, 43]. Different toxic drugs impair the activity of carnitine acylcarnitine translocase, which is an enzyme involved in the movement of fatty acids and production of energy across the inner membrane of cardiac mitochondria. ILEs may provide a sufficient amount of fatty acids to overcome drug intoxication induced fatty acid transport barricade and help in the restoration of the normal cardiac functions [39]. According to a third possible mechanism, ILEs enhance the intracellular level of calcium by directly activating voltage-gated calcium channels and thus ensuing in restoration of the myocyte activity. This property of ILEs is more valuable in those situations where calcium channel antagonist toxicity is prevailing [35, 44, 45].

Although 20 % ILEs are commonly used products with a safe track record for parenteral nutrition in humans, there are no clinical data available on the safety of short-term use of large boluses of these solutions [12]. The potential adverse reactions are usually associated with excessively high doses of ILEs and include thrombocytopenia, hemolytic anemia, jaundice, pancreatitis, hyperlipidemia, prolonged clotting time, hepato-splenomegaly, phlebitis and fat embolism [12, 35].

The optimal dose of ILEs for the treatment of ivermectin intoxication in lions is unknown. The initial dose of 1.5 ml/kg followed by a constant infusion of 0.25 ml/kg/min for 30 min was loosely based on the therapeutic recommendations of ILEs in humans. On the basis of these findings, it is rational to prescribe this dose of ILEs in veterinary medicine until additional studies recommend an optimal dosage schedule.

## Conclusion

The distinctive aspect of present case report is the intravenous use of a lipid emulsion in a lion for the management of ivermectin induced toxicity, which seemed to significantly truncate the course of recuperation.

## Consent

Consent was obtained from the owner of animal for publication of this case report.

## Abbreviations

VMTH: Veterinary medical teaching hospital
GABA: Gama-aminobutric acid
ILE: Intravenous lipid emulsion
GGT: Gamma glutamyl transferase
ALP: Alkaline phosphatase
TP: Total proteins
CRT: Capillary refill time
CNS: Central nervous system
MCTs: Medium-chain triglycerides
LCTs: Long-chain triglycerides
BUN: Blood urea nitrogen
RBCs: Red blood cells
PCV: Packed cell volume
Hb: Hemoglobin
